# Predictors of sentence recall performance in children with and without DLD: Complexity matters

**DOI:** 10.1017/S0305000924000345

**Published:** 2024-10-16

**Authors:** Janet L. McDonald, Janna B. Oetting

**Affiliations:** 1 Department of Psychology, Louisiana State University, Baton Rouge, Louisiana, USA; 2 Department of Communication Sciences & Disorders, Louisiana State University, Baton Rouge, Louisiana, USA

**Keywords:** Developmental language delay, sentence recall, nonword repetition, working memory, morphology

## Abstract

Using archival data from 106 children with and without DLD who spoke two dialects of English, we examined the independent contributions of vocabulary, morphological ability, phonological short term memory (pSTM), and verbal working memory (WM) to exact sentence recall, ungrammatical repetition, and incorrect tense production. For exact repetitions on simpler sentences, performance of the DLD group was predicted by morphological ability, pSTM and WM, while that of the TD group was predicted by vocabulary and sometimes pSTM. On complex sentences, performance of the DLD group was predicted by morphological ability, and the TD group was predicted by pSTM and WM. For ungrammatical repetitions and incorrect tense, morphological ability was a factor for both groups, with WM also affecting the DLD group for ungrammatical production. Thus, sentence recall taxes multiple resources, with more and different factors being taxed at lower levels of complexity for children with DLD than those without.

## Introduction

Children with developmental language disorder (DLD – formerly called specific language impairment or SLI) are known to have difficulty with sentence recall tasks relative to their typically developing (TD) counterparts. Sentence recall involves listening to an auditorily presented sentence, and attempting to repeat it back verbatim. Performance on such tasks has demonstrated both high sensitivity and specificity in classifying speakers across a variety of languages as having DLD or being TD (Archibald & Joanisse, 2009; Armon-Lotem & Meir, 2016; Conti-Ramsden et al., 2001; Leclercq et al., 2014; Oetting et al., 2016; Pham & Ebert, 2020; Redmond et al., 2019; Taha et al., 2021; Theodorou et al., 2017; Thordardottir et al., 2011; Tuller et al., 2018; Vang Christensen, 2019; Wang et al., 2022).

Sentence recall encompasses multiple types of linguistic knowledge and memory processes (Acha et al., 2021; Moll et al., 2015; Nag et al., 2018; Polišenská et al., 2015; Poll et al., 2013), including but not limited to a) vocabulary knowledge, b) morphological ability (as measured by picture matching tasks or morphology elicitation tasks), c) phonological short term memory (pSTM – being able to hold phonological information in store temporarily before giving it back, often measured by nonword repetition (NWR) tasks or forward digit or word spans), and d) working memory (WM – being able to hold on to and manipulate information, measured by tasks such as n-back or backward digit span). Compared to TD children, those with DLD have shown weaknesses in all four of these areas – vocabulary (e.g., Blom & Boerma, 2019; Ladányi & Lukács, 2019; McGregor et al., 2013), morphology (e.g., Leonard et al., 1992, 1999; Oetting et al., 2019, 2021; Vang Christensen & Hansson, 2012), pSTM (e.g., Delage & Frauenfelder, 2020; Dollaghan & Campbell, 1998; McDonald & Oetting, 2019; Montgomery & Evans, 2009) and WM (e.g., Frizelle & Fletcher, 2015; Ladányi & Lukács, 2019; McDonald et al., 2018; Montgomery & Evans, 2009). Studies have also found correlations between each of these factors and performance on sentence recall tasks in children with and without DLD (Alloway et al., 2004; Blom & Boerma, 2019; Moll et al., 2015; Poulsen et al., 2022; Riches, 2012 and the studies reviewed below). However, it is not clear if deficits in one or more of these factors lead to the poorer sentence recall performance in children with DLD compared to TD controls, or if these two groups rely on a different combination of these factors to recall sentences. It is therefore useful to determine which factors make independent contributions (i.e., account for additional variance in a regression) to sentence recall performance by children with and without DLD, to see which resources are being called upon to do the task. The goal of the current paper is to test the independent contributions of all four of the above delineated factors to sentence recall in each group. It would tell us different things about the nature of DLD if these profiles are similar but perhaps differing in strength (e.g., both groups are using both pSTM and WM, but more variance is accounted for in the DLD than the TD group), or if very different combinations of independent factors are involved in the two groups (e.g., the DLD group relies on pSTM and WM, while the TD group relies on vocabulary, indicating perhaps stress on the cognitive systems in the DLD group that is not happening in the TD group).

### Independent predictors of sentence recall

Prior studies have tested various subsets of these four predictors using regression to find the combination of the factors that independently best predicts sentence recall performance. A summary of all studies that tested two or more of these predictors in children using regression is presented in Table 1, with the age range of the children tested, if a first (L1) or second (L2) language was tested, and the type of test used to measure each factor. Some studies are in the table multiple times because of the different groups tested or the different combination of factors tested. The strength of each factor is given as a correlation when available, or otherwise as the beta or b weight from the regression. Significant contributors to the regressions are bolded and shaded in green.

For children with DLD, vocabulary was only a significant independent predictor in one of four cases where it was tested (Pratt et al., 2021 L1 speakers). In the other three cases, which all involved L2 speakers, it was either not a significant predictor or did not account for significant variance beyond WM (Pratt et al., 2021; Zebib et al., 2020). Morphological ability was correlated significantly to sentence recall performance in the three cases where it was tested (Hesketh & Conti-Ramsden, 2013; Zebib et al., 2020), but only added independent variance beyond pSTM or WM in one (Hesketh & Conti-Ramsden, 2013). Many studies examined pSTM through either NWR or forward digit span, and six of the analyses that included NWR as a pSTM measure found a significant correlation to sentence recall (but see Pratt et al., 2021 L1 speakers), and found it made an independent contribution beyond vocabulary (Pratt et al., 2021 L2 speakers), morphology (Hesketh & Conti-Ramsden, 2013) or WM (Delage & Frauenfelder, 2020; Ebert, 2014; Zebib et al., 2020; but see Vugs et al., 2016). Forward digit span as the sole measure of pSTM was not a good predictor (Zebib et al., 2020). All nine analyses that examined WM with other factors found it to make a significant independent contribution beyond vocabulary (Zebib et al., 2020), morphology (Zebib et al., 2020) or pSTM (Delage & Frauenfelder, 2020; Ebert, 2014; Vugs et al., 2016; Zebib et al., 2020). However, at least one study (not shown in Table 1 as it did not include regression across factors) failed to find a correlation between sentence recall and the WM measure of backward digit span for both monolingual and bilingual children with DLD (Talli & Stavrakaki, 2020).

For TD children, vocabulary was found to be a significant independent predictor in 13 regressions but not in one (Andreou et al., 2021 L1 speakers), accounting for variance beyond morphological ability, pSTM and/or WM (Acha et al., 2021; Andreou et al., 2021 L2 speakers; Monsrud et al., 2022; Nag et al., 2018; Pratt et al., 2021; Zebib et al., 2020). In the seven cases where it was tested, morphological ability was a significant independent predictor in regressions beyond vocabulary, pSTM or WM (Hesketh & Conti-Ramsden, 2013; Monsrud et al., 2022; Zebib et al., 2020), and a composite of vocabulary and morphological ability was an independent predictor beyond WM (Stadtmiller et al., 2022). Many studies examined pSTM, and it accounted for significant variance in 12 of 15 regressions – beyond vocabulary (Acha et al., 2021; Monsrud et al., 2022; Nag et al., 2018 word order; Pratt et al., 2021 L1 speakers; Zebib et al., 2020; but see Nag et al., 2018 inflections; Pratt et al., 2021 L2 speakers), morphology (Monsrud et al., 2022; Zebib et al., 2020; but see Hesketh & Conti-Ramsden, 2013) and WM (Delage & Frauenfelder, 2020; Zebib et al., 2020). WM, tested in 12 analyses, while usually correlated to sentence recall, made an independent contribution to five regressions beyond other factors (Acha et al., 2021 at second time measurement; Andreou et al., 2021 L1 speakers; Delage & Frauenfelder, 2020; Stadtmiller et al., 2022), but seven times it did not (Acha et al., 2021 at first time measurement; Andreou et al., 2021 L2 speakers; Zebib et al., 2020). In addition, Talli and Stavrakaki (2020 – again, not shown in the table as it did not do regressions) found nonword repetition (pSTM) and backward digit (WM) to be correlated with sentence recall for bilingual TD children, but only pSTM was correlated to sentence recall for monolingual children.

Looking at the summary of the studies in Table 1, two observations can be made. First, the information currently available is only suggestive of the independent importance of the four factors – vocabulary, morphological ability, pSTM and WM – as only one study (Zebib et al., 2020) has measured all four factors, and even this study did not enter all these factors into the same regression equation. The other studies tested two, or at most three, of the factors. Thus, there is a need to test all four predictors together to ascertain their independent contributions. Second, there were at least some discernible differences in factors that made independent contributions to sentence recall in children with and without DLD. Vocabulary was a strong independent predictor for TD children, but its role for children with DLD was less clear. Morphological knowledge, which perhaps has been understudied as contributor to sentence recall, seems to be a stronger independent predictor for TD children than for children with DLD. pSTM – especially when it included a measure of nonword repetition – was often, but not always an independent contributor in both groups. Finally, WM was a consistent independent contributor for the DLD groups but only sometimes contributed for the TD groups. The current study tests whether the patterns we noted for the DLD and TD groups in Table 1 hold up when all four predictors are entered into the same regression predicting sentence recall.

### The current study

The current study investigated the independent contributions of each of these four factors to the sentence recall of children with and children without DLD. The data were archival and included measures of vocabulary, morphology, pSTM, and WM as well as sentence recall performance from a group of children with DLD and a matched group of TD children who were speakers of two different dialects of English. Studies comparing these groups on each of these measures have been previously published (McDonald & Oetting, 2019; McDonald et al., 2018; Oetting et al., 2016, 2019); these prior studies all found differences between the DLD and TD groups within both dialects. In this study, we performed stepwise regression to see which of the four predictors made independent contributions (i.e., accounted for additional variance) to sentence recall performance in the DLD and then in the TD group. Based on the above literature review, we expected that the four predictors of vocabulary, morphology, pSTM, and WM would generally be correlated to exact sentence recall performance in both groups of children. However, we expected different patterns of factors would independently add to the regression in the children with and without DLD. Specifically, our predictions, in line with the findings in Table 1, were as follows:

1. for children with DLD, we predicted a particularly strong role for WM, with pSTM accounting for additional variance. Vocabulary and morphological ability may or may not account for additional variance.
2. For the TD children, we predicted independent contributions of vocabulary and morphological ability; pSTM may or may not add additional explained variance. WM should be the least likely to make an independent contribution for this group.

In addition, the contributions of the four predictors to sentence recall may differ depending on the nature of the sentences in the task. Indeed, studies have shown that repetition of syntactically complex sentences was correlated to WM, while that of simpler sentences was not (Delage & Frauenfelder, 2020; Frizelle & Fletcher, 2015). We might also expect effects of WM in children with DLD at a lower level of complexity than for TD children (Montgomery & Evans, 2009). The sentences we used in our sentence recall task varied in syntactic complexity based on number of functional categories they contained, with all sentences including the tense functional category, an area known to be difficult for children with DLD. Thus, we will be able to examine if complexity affected the factors involved in recall performance. We therefore predicted:

- WM will be more likely to be an independent contributor as sentence complexity increases, and children with DLD will show a stronger contribution of WM at lower levels of sentence complexity than the TD children.

Finally, most sentence recall studies have used exact repetition or a general scoring of repetition correctness as their main measure. However, other aspects of sentence recall also differentiate children with and without DLD. For example, children with DLD are more likely than TD children to produce an ungrammatical sentence (Gavarró, 2017; Oetting et al., 2016; Smolík & Matiasovitsová, 2021; Vang Christensen, 2019), and are more likely to make errors on tense (Oetting et al., 2016; Vang Christensen, 2019; Vang Christensen & Hansson, 2012). Tense errors have been shown to be correlated to vocabulary and pSTM in TD children (Nag et al., 2018) or across groups of DLD and TD children (Vang Christensen & Hansson, 2012). We therefore also examined the contributions of the four predictors to ungrammatical recalls and incorrect tense production in our sentence recall task. However, since prior work is scant, this aspect of our study is more exploratory.

- Specifically, we explored if the same pattern of factors predicted ungrammatical utterances and incorrect tense as had predicted the children’s correct sentence recall performance.

## Method

### Participants

Participants were 106 kindergarteners from rural schools in Louisiana who spoke one of two dialects – African American English (AAE) or Southern White English (SWE)^1^; 53 participants were children with DLD (18 SWE speakers; 35 AAE speakers) and 53 were typically developing children (18 SWE speakers; 35 AAE speakers). Classification into clinical group (DLD vs. TD) was made based on a review of standardized test scores and their language histories supplied by their families and their schools. Particularly, children in the DLD group scored ≤ −1 SD of the norm on a standardized test of syntax, the *Diagnostic Evaluation of Language Variation: Norm Referenced* (DELV-NR; Seymour et al., 2005), while the TD children scored above this criterion. In addition, all children scored ≥ −1.2 SD on the *Primary Test of Nonverbal Intelligence* (PTONI; Ehrler & McGhee, 2008) and ≥ −1 SD on the *Goldman-Fristoe Test of Articulation-Second Edition* (GFTA-*2*; Goldman & Fristoe, 2000). Forty-five percent of the children with DLD had a positive family history for language issues; this rate was 15% for the TD children. Dialect classification was based on blind listener judgments (see Oetting et al., 2016 for details). All speakers of AAE were African-American, and all speakers of SWE were not African-American. Children with DLD were matched to TD children within dialect for age, nonverbal intelligence scores and to the extent possible, maternal education.

Characteristics of the groups are given in Table 2. In 2 (clinical group) × 2 (dialect) ANOVAs, there were no significant differences by group or dialect for age or PTONI scores; there was a small group difference for maternal education favoring the TD group, but the effect size (η_p_^2^ = .05) was small. As expected, there were significant group differences on the DELV-NR test; there were also group differences as well as dialect differences on the GFTA favoring the TD group and the SWE speakers.

### Tasks

#### Vocabulary

To measure receptive vocabulary, all children completed the *Peabody Picture Vocabulary Test-4* (PPVT; Dunn & Dunn, 2007). As shown in Table 2, vocabulary scores differed by clinical group, with the TD children outperforming those with DLD; this effect size was large. As noted in the literature review, vocabulary weakness in children with DLD is a frequent and expected finding. Indeed, consonant with their DLD classification, 62% of this group had vocabulary scores under one standard deviation below the normed mean of 100, while none of the TD children did. There was also a main effect of dialect, with the SWE-speaking children having slightly higher vocabulary scores than AAE-speaking children.

#### Morphology

Morphology was tested using elicitation probes. Forms for past tense (e.g., *mowed, drew*), third person singular (e.g., *sees, saws*), auxiliary BE present (i.e., *is, are hugging*) and auxiliary BE past (i.e., *was, were building*) were elicited by showing children videos of an action accompanied with a prompt and then asking them to describe the actions (Oetting et al., 2019). For example, a video showing a man gluing a circle on a piece of paper was accompanied by the prompt, *The man doesn’t glue a square. The man doesn’t glue a triangle*, which was designed to elicit from the child, *The man glues/glue a circle*. Each structure was elicited with 16 different verbs, with a total of 64 different verbs across structures. Responses were recorded and then classified as an overt mainstream form (e.g., *drew*), an overt nonmainstream form (e.g., *drawed*, *had draw*), a zero form (e.g., *drawØ*), or other (e.g., *that’s pretty*). Examples of each of these form types can be found in Oetting et al. (2019). Proportion of overt forms was calculated by adding the children’s mainstream and nonmainstream overt forms and dividing by the overt forms plus the zero forms. As shown in Table 2, children with DLD produced lower proportions of overt forms than the TD children, with the effect size being large. Sensitivity of this test was good, with 72% of the children with DLD falling below a cut point determined by a discriminant analysis presented in Oetting et al. (2019). There was also an effect of dialect with lower proportions of overt forms by the AAE speakers; this finding was expected. Although both dialects allow zero forms as well as overt nonmainstream forms, zero forms are more frequent in AAE (Oetting & McDonald, 2001).

#### pSTM

We used the NWR task of Dollaghan and Campbell (1998) as our measure of pSTM (McDonald & Oetting, 2019). There were sixteen nonwords total – four each at 1, 2, 3 and 4 syllables in length. Recordings of the words were played and children were instructed to repeat each item out loud. Responses were recorded, and then scored for proportion of phonemes correctly produced. As shown in Table 2, the children with DLD had poorer NWR scores than the TD children; the effect size was large. Sensitivity was good on this measure, with 77% of the children with DLD falling below a cut point determined by a discriminant analysis presented in McDonald and Oetting (2019). The worse performance by the children with DLD was true within both dialects although there was a tendency for the difference to be larger for the speakers of SWE, especially with longer nonwords (McDonald & Oetting, 2019).

#### WM

Our measure of WM was a size judgment task (McDonald et al., 2018). In this task a list of concrete nouns was given, and the children had to reorder them in terms of size from smallest to largest before giving them back. There were three lists each consisting of 2, 3 and 4 words. We counted the number of correct small to large links that children produced across the nine lists. For example, if the list to reorder was *‘rabbit, bike, tooth’* and the child said *‘tooth, bike, rabbit’* they would earn one link for tooth to bike, but none for bike to rabbit. This measure thus captured both saying the correct words and saying them in the correct reordering; maximum score was 18 links. As shown in Table 2, the children with DLD had lower size judgment scores than the TD children; effect size was large. Sensitivity was good, with 77% of the children with DLD falling below a cut point determined by a discriminant analysis presented in McDonald et al. (2018).

#### Sentence recall

The sentence recall task had children repeat 36 different sentences (Oetting et al., 2016). There were 12 sentences at each of three levels of syntax complexity, depending on the number of functional categories involved (Hegarty, 2005). One functional category sentence involved tense (e.g., *Minnie is cleaning the dirty dishes in the sink*); two functional categories involved tense and negation (e.g., *Minnie* *is not* *cleaning the dishes in the sink*); and three functional categories involved tense, negation and complementizers (e.g., *Mickey wonders* *if* *Minnie* *is not* *cleaning the dishes*). Note that all sentences contained the tense functional category – a category children with DLD are known to find difficult. All sentences were 9 words in length, except for four of the three functional category sentences that were 7 words in length. The 12 sentences at each functional category level had three uses each of *is*, *are*, *was* and *were*.

Children’s productions were classified as exact repetitions, grammatical but not exact repetitions, ungrammatical repetitions (which includes tense and other errors), unscorable, or missing. Contracted forms of *is* and *are* were counted as exact repetitions as were productions of *is* and *was* in target sentences containing *are* and *were* due to their acceptability in the dialects. For example, if the target sentence was *Bert and Ernie are singing a new rap song, Bert and Ernie is singing a new rap song* was counted as an exact and grammatical recall. There were also 8 target sentences with third person verbal -s forms in an introductory clause (*Mickey wonders if…*); zero forms of this (e.g., *wonderØ*) within the children’s recalls were also counted as an exact and grammatical recall given the high frequency of these forms in AAE. Although permitted in both dialects, all other tense zero forms (e.g., *He Ø not jumping on the bed*) was counted as ungrammatical to capture the well documented finding that children with DLD produce higher frequencies of zero forms relative to same dialect-speaking TD groups (for repeated evidence for this claim, see Garrity & Oetting, 2010; Oetting et al., 2019, 2021; Oetting & McDonald, 2001).

Once scored, we divided exact repetitions, ungrammatical repetitions and incorrect tense by scorable items to get corresponding proportions. As shown in Table 2, compared to TD children, those with DLD had a lower proportion of exact repetitions and a higher proportion of ungrammatical recalls and tense errors and all these effect sizes were large. There was an interaction between group and dialect for the ungrammatical utterances; the effect of group was significant for both AAE speakers; *F*(1,68) = 34.03, *p* < .001, η^2^ = .33, and SWE speakers, *F*(1,34) = 48.40, *p* <.001, η^2^ = .59), although larger in the latter group.

## Results

Stepwise regressions (entry criterion .05, deletion criterion .10) were run separately on children with and without DLD, with vocabulary scores, morphology scores, proportion phoneme correct on NWR (pSTM measure), and correct links in the size judgment task (WM measure) as continuous predictors, and dialect spoken as a categorical predictor. While we did not expect many differences due to dialect spoken, adding this as a predictor allowed us to test for it. Checks for multicollinearity were run, and tolerances were well above .20 and VIFs were well below 4. Data are presented as correlation coefficients with the factors that contributed independently to each regression indicated in bold in each table; details of each regression are given in the appendix.

### Exact repetition

Children with DLD showed significant correlations in the moderate to strong range for three of the five factors – morphology, pSTM and WM (see Table 3). Stepwise regression showed all three factors to be independent contributors, in total accounting for 39% of the variance in exact repetition scores.

The TD group had moderate, nearly identical significant correlations for all factors (including vocabulary) except dialect spoken. Stepwise regression with the TD group had WM as the only predictor, accounting for 9% of the variance. While no other factor entered, the significance level for both vocabulary (*p* = .08) and pSTM (*p* = .08) were close to the entry criterion of .05. Indeed, when the entry criterion was relaxed to .08, the stepwise regression model converged on vocabulary and pSTM as independent contributors, accounting for 17% of the variance. Given these two regression results, which factors were the best predictors for TD children were not clear. We explored this further by looking at regressions for each level of complexity for the exact repetitions.

#### Effects of increasing sentence complexity

Recall that sentences differed in complexity by number of functional categories; we will refer to each level by its number of functional categories. Table 4 gives the performance at each of the three levels of complexity broken down by clinical group and dialect, as well as the results of the 2 (group) × 2 (dialect) ANOVA run on each level. At all levels, the DLD group produced lower exact repetitions than the TD group; there was also an effect of dialect at the first level of complexity, with the AAE speakers having higher scores than the SWE speakers. As can also be seen in Table 4, as complexity increased, exact repetitions fell for both groups; DLD: *F*(2,104) = 17.79, *p* < .001, η_p_^2^ = .26; TD: *F*(1.78,92.57) = 105.60, *p* < .001; η_p_^2^ = .67. Bonferroni corrected post-hoc tests on each group showed that one and two functional category sentences were different from the three functional category sentences.

Table 5 gives the results of the regression analyses for each level of complexity, and shows that for the DLD group, morphology, pSTM and WM as well as dialect (because AAE speakers had higher exact repetitions) were all correlated to performance and made independent contributions at sentence complexity level 1, and together, they accounted for 44% of the variance in performance. At sentence complexity level 2, the same predictors minus dialect were also correlated to performance and made independent contributions, accounting for 35% of the variance. However, for sentence complexity level 3, while both morphology and nonword repetition were correlated to performance, only morphology made an independent contribution, accounting for 9% of the variance. Note that performance was getting close to floor for the DLD group at complexity level 3, and this may be causing restriction of range.

For the TD group, vocabulary, pSTM and WM all correlated to exact repetitions at sentence complexity level 1, with vocabulary and pSTM making independent contributions that accounted for 19% of the variance. At sentence complexity level 2, vocabulary and morphology were correlated to performance, but only vocabulary made an independent contribution, accounting for 12% of the variance. Finally, at sentence complexity level 3, morphology, pSTM and WM were correlated to performance, with pSTM and WM making independent contributions that accounted for 19% of the variance. Thus, as complexity increased, the role of vocabulary decreased for TD children, and WM became important – contributing significant independent variance at sentence complexity level 3.

An interesting comparison can be made of the patterns of correlations for the DLD group at levels 1 and 2, and those for the TD group at level 3. These all showed correlations for morphology, pSTM and WM. This finding suggests that those with DLD were taxed by multiple processes at lower levels of complexity compared to the TD group, who became similarly taxed at only the highest level of syntactic complexity.

### Ungrammatical productions

Table 6 gives the results for ungrammatical productions. For the DLD group, morphology and WM as well as dialect spoken (recall that AAE speakers had a lower number of ungrammatical productions) made independent contributions in the stepwise regression, explaining 39% of the variance. For the TD group, only morphology scores made an independent contribution, explaining 20% of the variance.

Thus, for both groups, morphological ability was an important predictor of ungrammatical productions – children who had low productivity scores on the morphology elicitation task produced a higher number of ungrammatical utterances. Interestingly, WM only contributed additional variance for the DLD group. This may indicate that those with DLD were reaching their WM limitations, and thus were not able to coordinate the knowledge and processes needed to make a grammatical production.

### Tense errors

Table 6 also presents the results for tense errors. For the DLD group, only morphology was a significant predictor of tense errors, accounting for 27% of the variance. For the TD group, only morphology scores entered the regression and this explained 14% of the variance. Thus, for both groups, their morphological systems were negatively related to their tense errors – a morphological process, and this was the only independent predictor.

## Discussion

We examined what factors made independent contributions to sentence recall performance in children with and without DLD. Our first hypothesis predicted that pSTM and WM would be independent contributors to exact repetition scores for the children with DLD, and that vocabulary and morphology might or might not add extra explained variance. The analysis across all sentence types indeed found that pSTM and WM along with morphology all contributed independently to sentence recall performance while there was no evidence that vocabulary predicted sentence recall. Breaking the sentences down into their three complexity types showed similar results at complexity levels 1 and 2 – i.e., that pSTM, WM and morphology were independent predictors. However, at complexity level 3, when performance was getting near floor, only morphology made an independent contribution. The independent contributions of both pSTM and WM to performance at complexity levels 1 and 2 for children with DLD echoes the findings of others – especially those that measured pSTM using an NWR task (Delage & Frauenfelder, 2020; Ebert, 2014; Zebib et al., 2020; but see Talli & Stavrakaki, 2020; Vugs et al., 2016). An independent contribution of morphology was also found by Hesketh and Conti-Ramsden (2013; but see Zebib et al., 2020). The lack of contribution of vocabulary for children with DLD was also found by others (Pratt, 2021 – L2 speakers; Zebib et al., 2020) but mismatched the findings of Pratt et al. (2021 – L1 speakers). Overall, these findings showed that children with DLD were calling on multiple resources to successfully give exact repetitions of fairly simple sentences – pSTM was being used to remember and give back the phonological information, WM was being used store and process the linguistic information, and morphological processes were also being recruited.

Our second hypothesis predicted that vocabulary, morphology and pSTM would make independent contributions to the exact repetition performance of the TD group and that WM may play less of a role for this group. When viewing all sentences at once, the picture was somewhat murky, as all factors were fairly uniformly correlated with sentence recall performance. When we broke the sentences down by complexity level we found support for vocabulary making independent contributions at levels 1 and 2, with pSTM making an additional contribution at level 1. This partially confirms our second hypothesis; however morphology did not make its expected additional independent contribution. The importance of vocabulary for TD children’s sentence recall was found by numerous prior studies (Acha et al., 2021; Andreou et al., 2021 – L2 speakers; Monsrud et al., 2022; Nag et al., 2018; Pratt et al., 2021; Stadtmiller et al., 2022; Zebib et al., 2020). Independent contributions by pSTM were also often found (Acha et al., 2021; Delage & Frauenfelder, 2020; Monsrud et al., 2022; Nag et al., 2018 – L1 word order; Pratt et al., 2021 – L1 speakers; Zebib et al., 2020); note however, that it was sometimes found that pSTM did not add additional explained variance to a regression (Hesketh & Conti-Ramsden, 2013; Nag et al., 2018 – L1 inflections; Pratt et al., 2021 – L2 speakers). Our failure to find an independent contribution of morphological ability to the performance of the TD group contradicts prior findings of the importance of morphological mastery for sentence recall in TD children (Hesketh & Conti-Ramsden, 2013; Monsrud et al., 2022; Stadtmiller et al., 2022; Zebib et al., 2020). However, two of these studies did not include a measure of vocabulary along with morphology in their regressions (Hesketh & Conti-Ramsden, 2013; Zebib et al., 2020), which would leave more variance available for morphology to absorb. At level 3, when sentences were significantly more challenging, a different pattern of factors was found to contribute independently to the performance of TD children--here we found pSTM and WM made independent contributions, and we discuss the contribution of WM as part of the next hypothesis below.

Our third hypothesis stated that the involvement of WM may vary by sentence complexity, and that it may be engaged at a lower level of complexity for the DLD group than the TD group. Confirming this hypothesis, we found WM engagement for the DLD group at levels 1 and 2, but it only made an independent contribution for the TD group at level 3. This finding may help to explain why some prior studies found (e.g., Delage & Frauenfelder, 2020; Stadtmiller et al., 2022) and others did not find (e.g., Andreou et al., 2021 – L2 speakers; Zebib et al., 2020) an independent contribution of WM to the sentence recalls of TD children. It may be that WM only plays a unique role for TD children if the sentence recall task uses highly complex structures that are challenging for the age of the child tested. Indeed, Delage and Frauenfelder (2020) mentioned that WM predicted performance on only their most complex sentences – those with two or more levels of embedding or object relatives.

Looking across the two groups, our results indicated that vocabulary made independent contributions to exact sentence recall performance only for TD children at lower levels of complexity, morphology for the children with DLD at all levels of complexity, while pSTM tended to make independent contributions for both children with DLD (levels 1 and 2) and TD children (levels 1 and 3), and WM for children with DLD at lower levels of complexity and TD children at the highest level. Thus, the two groups had different patterns of dependence on the predictors at the individual levels. However, the resemblance of the predictors for the DLD group at lower levels of complexity to the TD group at the highest level of complexity raises the possibility that similar factors are involved once children were stressed; this stress just happened at a lower level of complexity for children with DLD than for those without.

As a fourth topic, we also explored what predictors were implicated in producing ungrammatical utterances and incorrect tense in sentence recall, something little examined in prior literature. The pattern of results here was clear – morphological ability clearly predicted ungrammatical productions and tense errors in both groups. Thus, predictors of ungrammatical utterances and tense errors were not the same as the predictors for exact sentence recall. Further, while others have found significant correlations between vocabulary or pSTM and tense errors in sentence recall (Nag et al., 2018; Vang Christensen & Hansson, 2012), we did not find that in our study. Note that WM only added additional explained variance for ungrammatical utterances for the children with DLD. This may indicate that WM capacity was being overwhelmed by the demand of the sentence recall task for children with DLD, and their strained capacity resulted in them not only failing to make an exact repetition, but failing to make a grammatical repetition.

We had speakers of two different dialects of English in our pool of tested children. Only occasionally did dialect spoken correlate to sentence recall performance, indicating that by and large these findings generalize across these dialects. And these few differences may be due to the way we scored the tasks. Recall that in order to distinguish between children with and without DLD within each dialect, we counted zero forms of tense as an error even though these zero forms are permitted in the dialects, with zero forms occurring at a much higher rate in AAE than SWE (Oetting et al., 2019; Oetting & McDonald, 2001; Oetting et al., 2021). Because of this, the correlation of dialect spoken with tense errors by the TD group was likely a result of the AAE speakers producing more zero forms for tense.

Studies that have investigated the contribution of pSTM to sentence recall used a variety of tests to measure this, including forward digit span, word span and NWR tasks. Looking at the literature reviewed in Table 1, NWR seemed to be the more powerful predictor amongst these tasks. This may be because NWR measures the ability of a child to extract phonological regularities at a sublexical level from their language exposure and then use this to help them repeat the nonwords (G. Jones, 2016; Szewczyk et al., 2018), and thus is both a measure of phonological STM as well as linguistic knowledge. Therefore, one should be cautious in thinking this is purely a measure of pSTM ability divorced from language knowledge, and we acknowledge that for our study as well.

Similarly, studies that have investigated the contribution of WM to sentence recall have used a variety of tests including backward digit span, counting span, listening span, n-back tasks, tonal matching, visual-spatial WM tasks; the size judgment task was used in our study. It is possible WM tasks that involve verbal material (like backward digit, listening span or size judgment) may differ from those that use nonverbal material (like tonal matching or visual-spatial tasks) in predicting sentence recall, as the former also load on linguistic knowledge. It is interesting to note however, that the nonverbal WM tasks used by studies in Table 1 did often account for independent variance in regression on sentence recall performance.

### Clinical/Research implications

We found that WM independently contributed to exact repetition performance for children with DLD at lower levels of complexity, and for TD children at the highest level of complexity. In addition, WM contributed to ungrammatical productions only for children with DLD. These results indicated a greater involvement of WM, and involvement at lower levels of complexity for children with DLD. One must be cautious in imputing causality from correlational methods (Marshall, 2020; Riches, 2020). However, recent exciting work by Delage et al. (2021) has shown that a training program that involved practicing both pSTM tasks and WM tasks not only improved performance on these tasks for both children with and without DLD, but improvement also transferred to a sentence recall task involving complex syntax (e.g., object relatives and one degree of embedding) for those with DLD. The results of Delage et al. (2021), taken in conjunction with our findings, strengthen the implication that children with DLD have a WM impairment or overload that affects their ability to accurately repeat sentences and to formulate a grammatical sentence when exact repetition fails. Research examining how working memory training affects the linguistic performance of children with DLD should continue.

With increased complexity, the pattern of factors impacting accurate sentence recall in TD children resembled that of children with DLD at lower levels. This raises the possibility that similar processes are being engaged in the two populations – it is just that children with DLD are overwhelmed at a lower level of complexity than TD children. This could be because of more limited WM capacity in children with DLD, or that their capacity is being devoted to other lower level processes, leaving less left over for higher order language processing. This latter idea fits in with recent work by S. D. Jones and Westermann (2022). Rather than viewing WM per se as being deficient in children with DLD, S. D. Jones and Westermann (2022) note that WM may be overloaded in these children due to inadequate linguistic representations of words. They showed that degraded auditory input resulted in a simulation having difficulty in distinguishing between different words, and having higher uncertainty even when the correct word was picked. This could lead to WM capacity having to be used to process lexical information, leaving less left over for other processes. This idea of WM being overloaded rather than deficient would help explain Smolík and Matiasovitsová’s (2021) findings of poorer sentence recall in children with DLD than those without, despite the two groups having similar WM scores.

It is important that future research on sentence recall performance in children with and without DLD continue to systematically manipulate syntactic complexity in the sentences tested (e.g., Delage & Frauenfelder, 2020; Frizelle & Fletcher, 2015; Nag et al., 2018; Zebib et al., 2020). Complexity can be manipulated in various ways – via number of functional categories as we did here, or by varying sentence type or number of embeddings as others have done. One can then compare what processes are used at each level by each group, and can then further see, when each group is taxed (albeit at different levels) if similar processes are involved. One could also consider adding an external load manipulation in future studies as another way to add taxation.

Finally, our study along with other studies in the field that have tested both children with and without DLD (Delage & Frauenfelder, 2020; Hesketh & Conti-Ramsden, 2013; Pratt et al., 2021; Zebib et al., 2020) modelled each group separately. This allowed for comparisons to work of others who only tested one or the other of the groups. Future work may want to also incorporate direct tests of the usefulness of the individual predictors between the two groups to enhance group comparisons.

## Conclusions

This is the first study to examine the independent influences of vocabulary, morphology, pSTM, and WM on the performance of a sentence recall task by children with and without DLD. We also used sentences that varied in complexity by number of functional categories, with every sentence including a tense marker, a category that is known to be difficult for children with DLD. Different patterns of usage were evident in the two population for overall exact repetitions with children with DLD relying on more and different factors (morphological ability, pSTM and WM) than the TD children (vocabulary and sometimes pSTM) for simpler sentences. In general, children with DLD appeared to be more overwhelmed by processing demands, and needed to use more resources – including WM – than TD children.

## Figures and Tables

**Table 1.** Summary of studies regressing factors with sentence recall performance in a) children with DLD and b) TD children

Study	Age	Language tested	Vocabulary task	Morphology task	pSTM task		WM task		
a) Children with DLD									
Pratt et al. (2021)	6–10 yrs	L1	**Expressive vocab L1 r=.45, p<.05**		NWR r=.10, ns				
Pratt et al. (2021)	6–10 yrs	L2	Expressive vocab L2 r=.24, ns		**NWR r=.47, p<.05**				
Zebib et al. (2020) ^a^	5–9 yrs	L2	Receptive vocab r=.39, ns		Forward digit r=.38, ns		**Backward digit r=.67. p<.001**		
Zebib et al. (2020) ^a^	5–9 yrs	L2	Expressive vocab r=.50, p<.05		Forward digit r=.38, ns		**Backward digit r=.67. p<.001**		
Hesketh & Conti-Ramsden (2013)	11 yrs	L1		**Past tense elicitation r=.64, p<.001**	**NWR r=.54, p<.001**				
Zebib et al. (2020) ^a^	5–9 yrs	L2		Receptive morphosyntax r=.58, p<.01	Forward digit r=.38, ns		**Backward digit r=.67. p<.001**		
Zebib et al. (2020) ^a^	5–9 yrs	L2		Expressive morphosyntax r=.65, p<.01	Forward digit r=.38, ns		**Backward digit r=.67. p<.001**		
Vugs et al. (2016)	4–5 yrs	L1			Forward digit, forward word, NWR – composite r=.38, p<.01		**Listening span, Counting span, backward digit – composite r=.45. p<.01**		
Ebert (2014)	5–11 yrs	L1			**NWR r=.35. p<.05**		**Tonal matching (nonverbal task) r=.41. p<.01**		
Ebert (2014)	5–11 yrs	L2			**NWR r=.38. p<.01**		**Tonal matching (nonverbal task) r=.33. p<.05**		
Delage & Frauenfelder (2020)	5–15 yrs	L1			**Forward digit, word span, NWR – composite β=.96, p<.001**		**Backward digit, counting span, running span – composite β=.61, p<.001**		
Zebib et al. (2020) ^a^	5–9 yrs	L2			**NWR r=.60, p<.01**	Forward digit r=.38, ns	**Backward digit r=.67. p<.001**		
b) TD children									
Monsrud et al. (2022)	6–13 yrs	L1	**Expressive vocab L1 β=.52, p<.001**	**Receptive morphosyntax L1 β=.14, p<.001**	**Word span L1 β=.23, p<.001**				
Monsrud et al. (2022)	6–13 yrs	L2	**Expressive vocab L2 β=.30, p<.001**	**Receptive morphosyntax L2 β=.20, p<.001**	**Word span L2 β=.25, p<.001**	**Word span L1 β=.13, p<.001**			
Stadtmiller et al. (2022) ^b^	5 yrs	L1	**Receptive vocab and morphosyntax composite L1 b=−.25. p<.001**				**N back b=−.41, p<.05**		
Stadtmiller et al. (2022) ^c^	5 yrs	L2	**Receptive vocab and morphosyntax composite L2 b=−.71, p<.001**				**N back b=−1.62, p<.05**		
Nag et al. (2018) ^d^	5–8 yrs	L1–word order	**Explain word meaning r=.50, p<.001**		**NWR r=.47. p<.001**				
Nag et al. (2018) ^d^	5–8 yrs	L1–inflections	**Explain word meaning r=−.38, p<.001**		NWR r=−.24, p<.01				
Pratt et al. (2021)	6–11 yrs	L1	**Expressive vocab L1 r=.52, p<.001**		**NWR r=.24, p<.05**				
Pratt et al. (2021)	6–11 yrs	L2	**Expressive vocab L2 r=.62, p<.001**		NWR r=.27, p<.01				
Zebib et al. (2020) ^a^	5–9 yrs	L2	**Receptive vocab r=.54, p<.001**		**Forward digit r=.49, p<.001**		Backward digit r=.41. p<.01		
Zebib et al. (2020) ^a^	5–9 yrs	L2	**Expressive vocab r=.66, p<.001**		**Forward digit r=.49, p<.001**		Backward digit r=.41. p<.01		
Acha et al. (2021)	6.4 yrs (Time 1)	Dominant lg	**Receptive vocab r=.48, p<.01**		**NWR r=.32, p<.01**		Backward digit r=.14, ns		
Acha et al. (2021)	7.5 yrs (Time 2)	Dominant lg	**Receptive vocab r=.41, p<.01**		**NWR r=.24, p<.01**		**Backward digit r=.29, p<.01**		
Andreou et al. (2021)	8–12 yrs	L1	Expressive vocab L1 r=.23, p<.05				Backwards digit r=.06, ns	**N–back r=.53, p<.01**	**Visualspatial r=.29, p<.05**
Andreou et al. (2021)	8–12 yrs	L2	**Expressive vocab L2 r=.28, p<.05**				Backwards digit r=.05, ns	N–back r=.12, ns	Visualspatial r=.05, ns
Hesketh & Conti-Ramsden (2013)	11 yrs	L1		**Past tense elicitation r=.53, p<.001**	NWR r=.43, p<.001				
Zebib et al. (2020) ^a^	5–9 yrs	L2		**Receptive morphosyntax r=.61, p<.001**	**Forward digit r=.49, p<.001**		Backward digit r=.41. p<.01		
Zebib et al. (2020) ^a^	5–9 yrs	L2		**Expressive morphosyntax r=.61, p<.001**	**Forward digit r=.49, p<.001**		Backward digit r=.41. p<.01		
Delage & Frauenfelder (2020)	5–13 yrs	L1			**Forward digit, word span, NWR – composite β=1.13, p<.001**		**Backward digit, counting span, running span – composite β=1.16, p<.001**		
Zebib et al. (2020) ^a^	5–9 yrs	L2			**Word repetition r=.65, p<.001**	**Forward digit r=.49, p<.001**	Backward digit r=.41. p<.01		

*Note:* NWR=nonword repetition. Factors that account for independent variance in the regression are bolded and have their cells shaded in green; significance of each factor is given after the correlation coefficient or regression weight.

a Zebib et al. (2020) did multiple regressions including different combinations of 3 variables each time.

b Stadtmiller et al. (2022) for omissions in the last part of the sentence.

c Stadtmiller et al. (2022) for omissions in the middle part of the sentence.

d Nag et al. (2018) scored recall accuracy at two different levels – one for word order and the other for inflections.

**Table 2.** Scores on demographics, standardized tests, predictors and sentence recall by group and dialect

	DLD						TD						Significant effects
	AAE n=35			SWE n=18			AAE n=35			SWE n=18			
	Mean	SD	*range*	Mean	SD	*range*	Mean	SD	*range*	Mean	SD	*range*	
Demographics													
Age (months)	66.94	3.74	*61–74*	65.72	3.89	*60–71*	65.60	3.55	*60–71*	66.61	4.17	*59–74*	none
Maternal Education	11.67	2.27	*6–17*	12.33	2.87	*8–17*	13.27	2.63	*8–17*	13.17	3.05	*6–17*	group: F(1,98)=4.96, p= .03, η^2^= .05
Standardized tests													
PTONI	93.69	9.62	*82–125*	96.50	8.35	*84–112*	98.09	8.87	*84–117*	98.28	8.14	*84–114*	none
DELV–NR Syntax	4.83	1.01	*3–7*	4.78	1.67	*1–7*	10.00	1.55	*8–14*	10.39	1.72	*8–14*	group: F(1,102)=328.80, p<.001, η^2^= .78
GFTA–2	104.49	5.72	*89–113*	104.78	4.18	*98–112*	107.00	4.38	*92–113*	110.50	3.09	*105–116*	group: F(1,102)=18.49, p<.001, η^2^= .15
													dialect: F(1,102)=3.92, p= .05, η^2^= .04
Predictors													
PPVT–4	83.34	9.42	*66–111*	85.78	7.01	*72–101*	101.06	9.32	*85–117*	105.56	5.62	*89–113*	group: F(1,102)=122.32, p<.001, η^2^= .54
													dialect: F(1,102)=5.20, p= .03, η^2^= .05
Morphology probe	.43	.22	*.06–.92*	.48	.30	*.05–.93*	.71	.20	*.35–1.00*	.91	.10	*.66–1.00*	group: F(1,102)=63.62, p<.001, η^2^= .38
													dialect: F(1,102)=7.82, p= .006, η^2^= .07
Nonword repetition	.70	.13	*.42–.92*	.63	.09	*.42–.79*	.80	.08	*.59–.94*	.80	.06	.67–.90	group: F(1,102)=46.47, p<.001, η^2^= .31
WM links	6.80	2.82	*1–13*	6.50	1.92	*3–10*	9.29	3.27	*1–14*	11.00	2.92	*5–17*	group: F(1,102)=35.25, p<.001, η^2^= .26
Sentence recall measures													
Exact repetition	.21	.14	*.00–.48*	.14	.14	*.00–.46*	.53	.18	*.00–.83*	.54	.20	*.20–.89*	group: F(1,102)=113.13, p<.001, η^2^= .53
Ungrammatical	.42	.19	*.17–.88*	.51	.23	*.17–.90*	.19	.13	*.00–.72*	.11	.08	*.00–.28*	group: F(1,102)=84.73, p<.001, η^2^= .45
													GxD: F(1,102)=6.46, p= .01, η^2^= .06
Incorrect Tense	.27	.21	*.08–.79*	.32	.25	*.06–.83*	.08	.11	*.00–.58*	.02	.02	*.00–.08*	group: F(1,102)=48.35, p<.001, η^2^= .32

DLD=Developmental Language Disorder; TD=Typically Developing; AAE=African American English; SWE=Southern White English; Maternal Education is based on grade level completed, with 17 indicating schooling beyond undergraduate; PTONI=Primary Test of Nonverbal Intelligence (standardized to have mean of 100 and SD of 15); DELV-NR= Diagnostic Evaluation of Language Variation-Norm Referenced Syntax score (a score of 7 is 1 SD below the mean); GFTA=Goldman-Fristoe Test of Articulation (standardized to have mean of 100 and SD of 15); PPVT=Peabody Picture Vocabulary Test (standardized to have mean of 100 and SD of 15); Morphology probe is a proportion correct production score; Nonword repetition is proportion phoneme correct score; WM links could range from 0 to 18; Exact repetition is the proportion of scorable sentences correctly repeated; Ungrammatical sentence recall is proportion of scorable sentences that were ungrammatical; incorrect tense recall is proportion of scorable sentences containing a tense error; GxD=group by dialect interaction.

**Table 3.** Correlations between predictors and exact repetition in sentence recall performance

	Vocabulary	Morphology	Nonword	Working	Dialect
			Repetition	Memory	
Exact repetition					
DLD	.18	**.41** **	**.50** ***	**.44** ***	−.24
TD	**.31** * ^b^	.30*	**.28** * ^b^	.31*^a^	.03

Note:

* p<.05;

** p<.01;

*** p<.001.

significant contributors to the regression in **bold and shaded in green**.

a stepwise regression had working memory as the sole factor.

b stepwise regression with relaxed entry criterion had vocabulary and nonword repetition as independent factors.

**Table 4.** Exact repetitions for each sentence complexity level by group and dialect

	DLD				TD				Significant effects
	AAE n=35		SWE		AAE n=35		SWE n=18		
	Mean	SD	Mean	SD	Mean	SD	Mean	SD	
Level 1	.27	.19	.12	.14	.63	.22	.59	.22	Group F(1,102)=105.63, p<.001, η^2^=.51
									Dialect F(1,102)=5.46, p=.02, η^2^=.05
Level 2	.25	.20	.19	.22	.62	.20	.65	.23	Group F(1,102)=93.74, p<.001, η^2^=.48
Level 3	.11	.09	.10	.12	.39	.19	.34	.20	Group F(1,102)=65.42, p<.001, η^2^=.39

**Table 5.** Correlations between predictors and exact repetition by complexity level

	Vocabulary	Morphology	Nonword Repetition	Working Memory	Dialect
DLD					
Level 1	.15	**.32** *	**.51** ***	**.43** ***	**−.39** **
Level 2	.15	**.41** **	**.45** ***	**.42** **	−.12
Level 3	.21	**.31** *	.27*	.18	−.04
TD					
Level 1	**.34** *	.23	**.28** *	.30*	−.08
Level 2	**.35** **	.27*	.20	.22	.05
Level 3	.11	.32*	**.33** *	**.34** *	.14

Note:

* p<.05;

** p<.01;

*** p<.001.

significant contributors to the regression in **bold and shaded in green**.

**Table 6.** Correlations between predictors and ungrammatical productions and tense errors in sentence recall performance

	Vocabulary	Morphology	Nonword Repetition	Working Memory	Dialect
Ungrammatical productions					
DLD	−.07	**−.49** ***	−.38**	**−.40** **	.22
TD	−.33*	**−.44** ***	−.16	−.25	−.30*
Tense errors					
DLD	−.01	**−.52** ***	−.26	−.25	.11
TD	−.24	**−.38** **	−.13	−.16	−.27*

Note:

* p<.05;

** p<.01;

*** p<.001.

significant contributors to the regression in **bold and shaded in green**.

## Footnotes

1 Exploring DLD within these dialects is challenging because some features of the dialects – for example zero marking of tense and agreement – overlap with output often produced by both children with and without DLD. Thus, a child might say *He Ø walking* or *She seeØ the dog* because zero forms of these morphemes are grammatical in their dialect, or because they have DLD and are relying on zero forms at dialect- and age-inappropriate frequencies. We have little reason to expect differences in TD sentence recall based on dialect spoken (Oetting et al., 2016). Nevertheless, we added dialect spoken as a factor in our regression to test for any effects. AAE speakers produce higher rates of zero forms than SWE speakers (Oetting & McDonald, 2001), which may result in dialectal differences when we specifically examine the children’s marking of tense. However, as specified in the methods, we strategically scored the recalls, accepting the children’s zero forms of third person verbal -s as correct in their recalls given the ubiquity of this form in AAE (Cleveland & Oetting, 2013; Newkirk-Turner & Green, 2021).

## Appendix: Details of stepwise regressions

**Table:**

	R^2^	beta	t	p
Stepwise regression DLD exact repetition				
NWR	.25	.32	2.60	.012
Morphology	.34	.29	2.49	.016
WM	.39	.25	2.04	.047
Stepwise regression TD exact repetition				
WM	.09	.31	2.30	.026
Stepwise regression TD exact repetition with relaxed entry criterion				
WM	.09			
Vocabulary	.15	.30	2.30	.026
NWR	.20	.27	2.115	.039
WM removed	.17			
Stepwise regression DLD exact repetition level 1				
NWR	.26	.25	1.97	.054
Dialect	.32	.33	2.86	.006
WM	.39	.26	2.21	.032
Morphology	.44	.24	2.15	.036
Stepwise regression DLD exact repetition level 2				
NWR	.20	.27	2.09	.042
Morphology	.30	.30	2.57	.013
WM	.35	.26	2.01	.050
Stepwise regression DLD exact repetition level 3				
Morphology	.09	.31	2.30	.026
Stepwise regression TD exact repetition level 1				
Vocabulary	.12	.33	2.62	.012
NWR	.19	.27	2.12	.039
Stepwise regression TD exact repetition level 2				
Vocabulary	.12	.35	2.70	.009
Stepwise regression TD exact repetition level 3				
WM	.11	.29	2.22	.031
NWR	.19	.28	2.13	.038
Stepwise regression DLD ungrammatical				
Morphology	.24	−.45	−3.97	<.001
WM	.33	−.30	−2.66	.011
Dialect	.39	−.24	−2.19	.034
Stepwise regression TD ungrammatical				
Morphology	.20	−.44	−3.53	<.001
Stepwise regression DLD tense errors				
Morphology	.27	−.52	−4.36	<.002
Stepwise regression TD tense errors				
Morphology	.14	−.38	−2.93	.005
